# 

**Published:** 1888-12

**Authors:** 


*Now is the time to Renew Your Subscription.*

Vol. IX. DECEMBER, 1888. No. 12.

LIBRARY
INDEXED
S.G.O.

### THE Independent Practitioner:

### A MONTHLY JOURNAL

DEVOTED TO

### DENTAL AND ORAL SCIENCE.

---

W. XAVIER SUDDUTH, M. D., D. D. S.,

EDITOR.

---

THE INTERNATIONAL DENTAL JOURNAL ASSOCIATION,

PUBLISHERS,

51 West 37th Street, New York City,

AND

1215 Filbert Street, Philadelphia.

**$2.50 Per Annum in Advance, - - - - Single Copies, 25 Cents.**

Entered at the Post-Office, Philadelphia, Pa., as Second-Class matter.

